# Characterization and Discrimination of Volatile Compounds of Donkey and Horse Meat Based on Gas Chromatography–Ion Mobility Spectrometry

**DOI:** 10.3390/foods14071203

**Published:** 2025-03-29

**Authors:** Yan Zhao, Xinyi Du, Shuang Liu, Mengqi Sun, Limin Man, Mingxia Zhu, Guiqin Liu, Muhammad Zahoor Khan, Changfa Wang, Mengmeng Li

**Affiliations:** School of Agriculture and Biology, School of Materials Science and Engineering, Liaocheng Research Institute of Donkey High-Efficiency Breeding and Ecological Feeding, Liaocheng University, Liaocheng 252000, China; 16652288780@163.com (Y.Z.); duxinyi1289@163.com (X.D.); luyueshuang1214@163.com (S.L.); 17862555726@163.com (M.S.); lmman@lcu.edu.cn (L.M.); zhumingxia@lcu.edu.cn (M.Z.); guiqinliu@lcu.edu.cn (G.L.); zahoorkhattak91@yahoo.com (M.Z.K.)

**Keywords:** donkey meat, horse meat, gas chromatography–ion mobility spectrometry, volatile compounds, flavor, multivariate analysis

## Abstract

The production of high-quality specialty meats has emerged as a prominent research focus within the livestock industry, under the broader concept of big food. However, the composition and variances of volatile compounds (VOCs) in donkey meat (DM) and horse meat (HM) remain unclear, which complicates their effective identification. In the present study, the VOCs of DM and HM were analyzed using gas chromatography–ion mobility spectrometry (GC-IMS) in combination with a multivariate analysis. Our results indicate that a total of 39 VOCs were identified in both DM and HM. These VOCs were categorized into five groups: aldehydes (39.53%), ketones (28.89%), alcohols (28.89%), acids (6.98%), and furans (2.33%). Compared with HM, the concentration of aldehydes, ketones, and alcohols in DM is significantly higher. (*p* < 0.001). Additionally, 16 characteristic-flavor VOCs were identified in both types of meat, with notable compounds including oct-1-en-3-ol, 3-hexanone, and heptanol. Topography, fingerprinting, and multivariate analysis effectively differentiated the VOC profiles of DM and HM. Furthermore, the 28 differential VOCs identified in DM and HM were all significantly higher in DM than in HM. In summary, this study conducted a comprehensive analysis of the VOC composition and characteristic flavor compounds in DM and HM, highlighting key differential VOCs. These findings contribute valuable data for flavor regulation and offer technical support for detecting the adulteration of DM with HM. The difference in sensory quality between DM and HM needs further research.

## 1. Introduction

Meat has become a fundamental component of the human diet, being a rich source of high-quality protein, essential fatty acids, vitamins, and trace nutrients such as zinc, selenium, iron, and phosphorus [1]. With rising living standards, the demand for high-quality specialty meats has gained significant importance [2]. 

The adage “Dragon meat in the sky, donkey meat on the ground” reflects not only the palatability of donkey meat (DM), but also its unique nutritional profile [3]. DM is considered a luxury food due to its superior nutritional composition, including higher levels of protein, essential amino acids, and fatty acids, along with lower fat content, cholesterol, and caloric value [4]. However, DM remains relatively scarce and expensive in the marketplace, primarily due to factors such as the long growth cycle of donkeys, low population numbers, limited reproductive rates, and the availability of few high-quality breeds [5]. In contrast, horse populations are more abundant, with a wider variety of breeds and higher meat yield; however, HM is often characterized by coarser muscle fibers and a more pronounced, sour taste [6]. In recent years, incidents of DM adulteration have occurred periodically, typically involving the sale of HM as DM or the mixing of DM with HM for sale [7]. Consequently, there is an urgent need for reliable methods and techniques to differentiate DM from HM, which will be crucial for safeguarding the integrity of the donkey meat industry and supporting its sustainable development.

Freshness and sweetness are the characteristic flavors of DM [8], whereas HM exhibits a milder flavor with a slightly sour aftertaste [6]. These flavor differences can be attributed to variations in volatile organic compounds (VOCs) between DM and HM. Aldehydes, hydrocarbons, ketones, and alcohols are the primary VOCs present in both in DM and HM [9,10]. These compounds are produced using series of chemical reactions including Maillard reactions, lipid degradation, the thermal degradation of thiamine, and Maillard–lipid interactions [11]. Notably, approximately 90% of VOCs are produced through lipid degradation, with the remaining 10% originating from other reactions [10]. Lipids, which are a key component of meat nutrition [12], can be influenced by factors such as genetic selection, age at slaughter, geographical location, diet, and exercise [13]. Previous research has highlighted significant differences in lipid content and fatty acid composition between DM and HM [14]. Therefore, investigating the VOC profiles of DM and HM represents a promising approach to distinguish between these two types of meat.

The volatile flavor compounds produced in food are complex in composition, with relatively small molecular weights, low content, and instability. When extracting and analyzing the desired flavor compounds, appropriate methods should be selected. Gas chromatography–mass spectrometry (GC-MS) is particularly useful in food flavor analysis, but the complex preprocessing limits its application in rapid detection [15]. Gas chromatography–ion mobility spectrometry (GC-IMS) technology has become an emerging method for rapid detection of sample quality due to its speed, portability, and ease of operation [16,17]. GC-IMS has been successfully applied for food classification, freshness evaluation, spoilage detection, and the analysis of aroma changes during storage [13,18,19]. For example, significant differentiation of flavor volatiles from different parts of chicken slaughter and cutting processes was successfully achieved using GC-IMS and stoichiometry [20]. In this study, GC-IMS, combined with a multivariate analysis, was employed to investigate the composition and characteristic flavor compounds of VOCs in DM and HM, as well as to identify differences in their VOC profiles. The aim was to provide fundamental data for the flavor regulation of DM and HM and to offer technical support for detecting HM adulteration in DM.

## 2. Material and Methods

### 2.1. Ethical Statement, Sample Collection, and Processing

The experimental procedures were approved by the Animal Care and Use Committee of Liaocheng University (approval number: 2023022706). The experimental animals, consisting of 6 donkeys and 6 horses, were sourced from a local breeding farm in Liaocheng, Shandong. All animals were in good health and were managed and raised under identical conditions. They were of similar weight, about 2 years old, and were subjected to the same environmental conditions throughout the study. Following a 12 h fasting period in accordance with international guidelines (CAC/RCP41-1993 and ISO/TS34700:2016), the animals were transported to local slaughterhouses where they were humanely slaughtered. The longissimus dorsi muscles were subsequently collected, packaged, and rapidly frozen by immersion in liquid nitrogen. After transportation back to the laboratory, the samples were stored at −80 °C for subsequent GC-IMS testing.

### 2.2. GC-IMS Analysis of VOCs in DM and HM

A FlavourSpec (Flavourspec^®^-G.A.S. Dortmund, Germany Company) flavor analyzer equipped with capillary columns was used for the analysis of VOCs in DM and HM. The instrument was equipped with an automated sampling device, and 2.0 g of the meat sample was weighed accurately and placed in a 20 mL headspace glass vial with 0.5 μL internal standard substance of 2-methyl-3-heptanone (0.1 g/L) (Merck Life Science and Technology, Nantong, China Co., Ltd.). The sample was then incubated at 60 °C with continuous agitation at 500 rpm for 20 min. During the analysis, the carrier gas flow rate was initially set at 2 mL/min for the first 2 min. The flow rate was gradually increased to 100 mL/min over the next 6 min (from 2 to 8 min), followed by a further increase to 150 mL/min during the subsequent 12 min (from 8 to 20 min). The total duration of the process was approximately 20 min. Nitrogen gas with a purity >99.999% (Shandong, China Wanbang Gas Co.) was used as carrier and drift gases, with a flow rate set at 150 mL/min. The drift tube had a length of 9.8 cm, and the drift voltage was applied at 5 kV. Drift temperatures were maintained at 60 °C for the gas chromatographic separation and at 45 °C for ion mobility spectrometry analysis. Separation was carried out on a gas chromatograph equipped with an MXT-5 column (15 m × 0.53 mm × 1.0 µm). Positive ion mode was selected for ionization, with the ionization source energy set at 3 eV.

### 2.3. Identification of VOCs

A calibration curve for the retention time (RT) and retention index (RI) was established using a mixed standard of six ketones (2-butanone, 2-pentanone, 2-hexanone, 2-heptanone, 2-octanone, and 2-nonanone). The RIs of C4-C9 n-ketones (Sigma Aldrich (Shanghai) Trading Co., Ltd., Shanghai, China) obtained under the same experimental conditions were used to compare the RIs of VOCs. The RI and Dt of the standards from the NIST (National Institute of Standards and Technology, Gaithersburg, MD, United States) and GC-IMS database (G.A.S., Dortmund, Germany) were utilized to distinguish volatiles. Based on 2-methyl-3-heptanone internal standard, a semi-quantitative analysis was performed based on peak volume.

The formula for calculating odor activity values (OAV) is as follows:

OAVi = Ci/Ti × 100(1)

Ci and Ti represent the absolute content and corresponding sensory thresholds of each VOCs, respectively. Flavors with OAV > 1 are considered characteristic flavor compounds.

### 2.4. Statistic Analysis

Each sample was analyzed in six sets of parallel trials, and the data on VOCs in DM and HM were analyzed for differences using SPSS 24.0 (SPSS Inc., Chicago, IL, USA). Differences between samples were analyzed using Tukey’s test. The data were presented as mean ± standard error of mean (SEM), and *p* < 0.05 was considered significantly different. The Reporter plugin and the Gallery Plot plugin were utilized to construct spectral and fingerprint representations, respectively. MetaboAnalyst 5.0 online software was used for principal component analysis (PCA), partial least squares discriminant analysis (PLS-DA), orthogonal partial least squares discriminant analysis (OPLS-DA), and heat map visualization. Differential volatiles were determined using variable importance in projection (VIP) > 1 and *p* < 0.05.

## 3. Results

### 3.1. VOC Profiles of DM and HM

**Figure 1:**
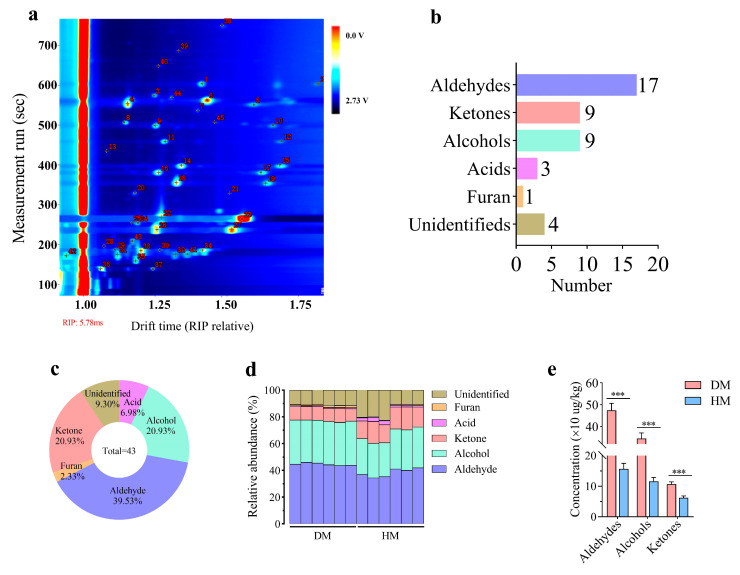
Volatile compounds in donkey and horse meat. Quantity of volatile compounds (**a**). The quantity (**b**) and percentage (**c**) of volatile compounds types. The percentage (**d**) and concentration (**e**) of volatile compounds in donkey and horse meat. The data are expressed as mean ± standard error (*n* = 6), *** *p* < 0.001. DM, donkey meat. HM, horse meat.

As shown in Figure 1 and Table 1, a total of 43 VOCs (17 aldehydes, 9 ketones, 9 alcohols, 3 acids, 1 furan, and 4 unidentified) were detected in DM and HM, including 39.53% aldehydes, 20.93% ketones, 20.93% alcohols, 6.98% acids, 2.33% furans, and 9.30% unidentified. Among them, 39 VOCs were identified in both DM and HM (Figure 1a–c). Aldehydes, alcohols, and ketones were the main categories of VOCs in both DM and HM (Figure 1d). Notably, the concentrations of aldehydes, alcohols, and ketones in DM were significantly higher than those in HM (*p* < 0.001; Figure 1e).

### 3.2. Characteristic Flavors

**Figure 2:**
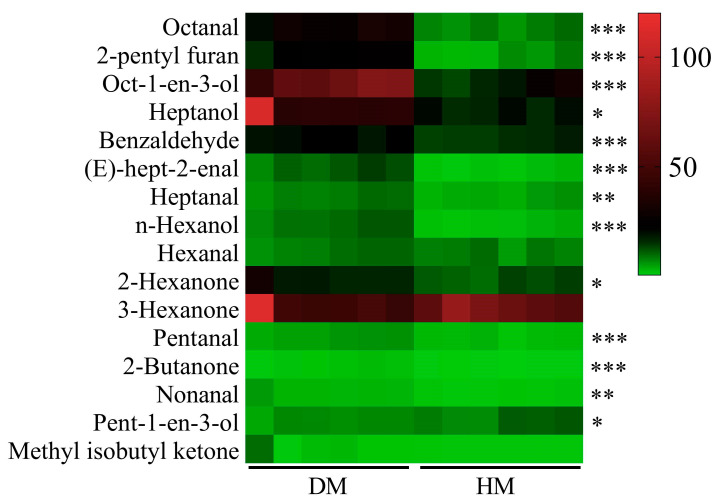
Characteristics of volatile flavor compounds in donkey and horse meat. Red expresses a high content of the flavor compound in the pattern, while green expresses a low content of the flavor compounds in the pattern. * *p* < 0.05, ** *p* < 0.01, *** *p* < 0.001. *n* = 6, DM, donkey meat. HM, horse meat.

As shown in Figure 2, a total of 16 characteristic volatile flavor compounds were identified based on an OAV > 1. These compounds include 3-hexanone, oct-1-en-3-ol, heptanol, octanal, 2-pentyl furan, benzaldehyde, 2-hexanone, pent-1-en-3-ol, hexanal, (E)-hept-2-enal, heptanal, n-hexanol, pentanal, 2-butanone, nonanal, and methyl isobutyl ketone. Notably, the OAVs of oct-1-en-3-ol, octanal, and 2-pentyl furan in DM were markedly higher than those measured in HM.

### 3.3. Difference in VOCs

**Figure 3:**
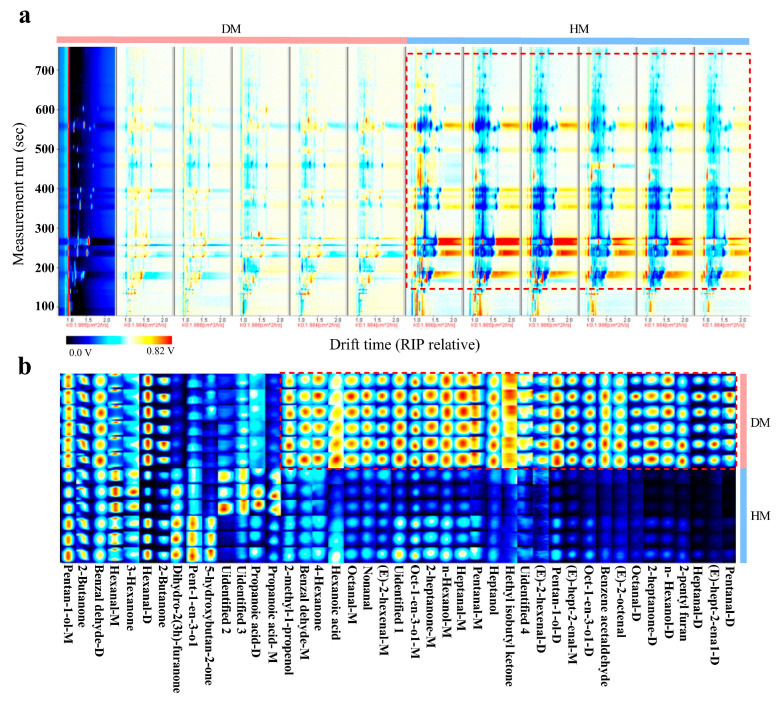
Differential volatile compounds in donkey and horse meat. Differential spectra (**a**) and fingerprint spectra (**b**) of volatile compounds. The color brightness of the signal peak is directly proportional to the concentration of the component, that is, the brighter the color, the higher the concentration of the component. *n* = 6, DM, donkey meat. HM, horse meat.

As shown in Figure 3, significant differences in fingerprint features between DM and HM can be observed (Figure 3a). Moreover, several compounds, including pentanal, (E)-hept-2-enal, heptanal, 2-pentyl furan, n-hexanol, 2-heptanone, octanal, (E)-2-octenal, benzene acetaldehyde, oct-1-en-3-ol, pentan-1-ol, (E)-2-hexenal, ethyl isobutyl ketone, heptanol, nonanal, hexanoic acid, 4-hexanone, benzaldehyde, and 2-methyl-1-propanol, exhibited distinct signals between DM and HM, suggesting that these compounds contribute to the differentiation of the VOC fingerprint profiles of DM and HM (Figure 3b)

### 3.4. Multivariate Analysis of VOCs

**Figure 4:**
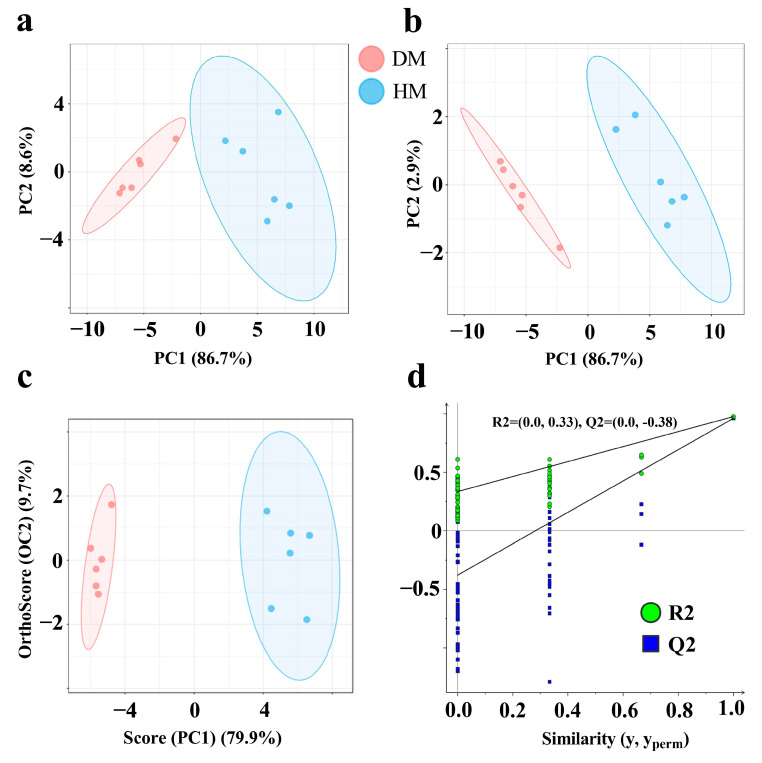
Multivariate analysis of volatile compounds in donkey and horse meat. Principal component analysis (PCA; (**a**)), partial least squares discriminant analysis (PLS-DA; (**b**)), and orthogonal partial least squares discriminant analysis (OPLS-DA; (**c**)) scoring charts; and the corresponding OPLS-DA validation diagram (**d**). *n* = 6, DM, donkey meat. HM, horse meat.

As shown in Figure 4, PCA successfully differentiated meat samples based on their VOCs (Figure 4a). This differentiation coincided with the results obtained from PLS-DA and OPLS-DA (Figure 4b,c). The intercept values for R^2^ and Q^2^ were (0, 0.33) and (0, −0.38), respectively. Notably, all Q^2^ values were lower than the original Q^2^ value, which is located at the far-right of the plot, and the Q^2^ regression line intersected the vertical axis at a value below zero (Figure 4d). This indicates that the OPLS-DA model demonstrates strong robustness and reliability, with no evidence of overfitting. These findings suggest that the distinct differentiation between DM and HM samples based on VOCs is achievable using a multivariate analysis. As Table 2 shows, a total of 28 differentially VOCs were identified in DM and HM based on VIP > 1 and *p* < 0.001, which including five categories: 14 aldehydes, 8 alcohols, 4 ketones, 1 furan, and 1 acid. The levels of (E)-2-hexenal, (E)-2-octenal, (E)-hept-2-enal, 2-butanone, 2-hexanone, 2-heptanone, 2-methyl-1-propanol, 2-pentyl furan, heptanal, heptanol, hexanal, nonanal, octanal, pentanal, benzene acetaldehyde, hexanoic acid, n-hexanol, oct-1-en-3-ol, and pentan-1-ol were markedly higher in DM than those measured in HM (*p* < 0.001).

## 4. Discussion

In this study, GC-IMS coupled with a multivariate analysis was employed to characterize VOC profiles in both DM and HM. A total of 39 VOCs, categorized into five distinct groups, were identified in both DM and HM. In contrast, a previous study reported 109 and 122 VOCs in DM using GC-MS [21,22], representing a substantially higher number of VOCs than those detected in the current study. The GC-IMS used in this study exhibits high sensitivity in the low parts per billion by volume (ppbv) range, making it highly effective for quantifying low-abundance VOCs. In contrast, GC-MS is more adept at detecting a broader spectrum of VOCs, but generally exhibits lower sensitivity with regard to low-concentration compounds [23,24]. Thus, the differences in VOC detection between these two analytical techniques likely contribute to the observed variance in the number of identified compounds. Aldehydes were found to be the predominant class of VOCs in both DM and HM, which is in line with the findings reported in previous studies on VOC profiles in these types of meat [9,25]. Notably, the concentrations of aldehydes, alcohols, and ketones were generally higher in DM compared to HM. This is consistent with prior research indicating that these VOCs are typically more concentrated in DM than in pork, bovine, or sheep meat [26], further suggesting that VOC profiles may vary significantly across different species and breeds of meat.

The VOCs are released from the surface of meat and are therefore closely related to its various sensory characteristics [27]. The impact of flavor compounds on the overall sensory properties of meat is determined not only by their concentration, but also by their OAVs, which reflect the potency of these volatiles in influencing the food’s aroma [28]. Compounds with OAVs ≥ 1 are typically considered to have a significant impact on flavor [29]. In this study, a total of 16 VOCs with OAVs ≥ 1 were the characteristic VOCs in DM and HM, mainly including 3-hexanone, oct-1-en-3-ol, heptanol, octanal, and 2-pentyl furan. Characteristic VOCs were identified in DM, including hexanal, 1-octen-3-ol, (E,E)-2,4-nonadienal, nonanal, octanal, dodecanal, (E,E)-2,4-decadienal, heptanal, 2-pentyl-furan, (E)-2-octenal, and 5-methyl-2-hexanone [10]. Among these, oct-1-en-3-ol, octanal, and 2-pentyl furan were considered key characteristic VOCs in both DM and HM. Notably, which imparts a mushroom- and fruit-like aroma, is a common and distinctive flavor compound in spiced beef [30]. Previous studies have also identified octanal as a characteristic VOC, associated with green, citrus, and lemon-like flavors in soy-sauce-marinated beef as well as in frozen white and red meats [31,32]. The aroma of 2-pentyl furan has been described as reminiscent of green bean and butter in chicken and duck meat [33,34]. These findings suggest that mushroom, green, and butter-like flavors are prominent in both DM and HM. Further analysis revealed that oct-1-en-3-ol, octanal, and 2-pentyl furan exhibited significantly higher OAVs values in DM compared to HM, whereas 3-hexanone displayed the opposite trend, with higher OAVs in HM. These results validated the sensory observations that mushroom, green, and butter flavors were more pronounced in DM, while grape and wine-like flavors were more prominent in HM.

The use of GC-IMS in combination with spectroscopy and fingerprint recognition technology provides an effective and intuitive approach for differentiating between various samples based on their VOC profiles [35]. This technology has achieved proven efficacy in several areas, including the classification of meat products, detection of freshness, and identification of adulterated meat [36,37]. The integration of fingerprinting technology offers a macroscopic and visual method for analyzing and comparing subtle spectral differences in VOCs between samples [38]. The effects of dietary roughage on VOC profiles in donkey milk were characterized using GC-MS [39]. A comparative analysis of VOC distribution across different DM fractions was conducted via GC-IMS [40]. Our investigation revealed statistically significant divergence in VOC spectral signatures between DM and HM, corroborating previous findings that demonstrated distinct VOC profiles across various chicken breeds [41]. Specifically, the signals for pentanal, (E)-hept-2-enal, and heptanal were significantly stronger in DM compared to HM. To validate the accuracy of these findings, a multivariate analysis was employed to confirm the data and fingerprint profiles derived from GC-IMS, with a particular focus on the differential VOCs [42]. The PCA and PLS-DA have proven effective in distinguishing between meat species such as chicken, duck, pork, bovine, and sheep [43,44]. In our study, VOCs were successfully differentiated between DM and HM using PCA, PLS-DA, and OPLS-DA, aligning with previous findings for species differentiation in chicken, chevon, beef, and DM [45]. A total of 28 differentially VOCs were identified in DM and HM, including compounds such as (E)-2-hexenal, (E)-2-octenal, (E)-hept-2-enal, nonanal, and heptanal, which are consistent with the fingerprint analysis results. (E)-2-hexenal has become a key volatile compound for identifying different lamb breeds [46]. And the content of nonanal and (E)-2-octenal has become an important indicator for identifying whether duck meat is fresh [47]. Notably, (E)-2-octenal and nonanal concentrations were higher in DM compared to HM. These substances are recognized as oxidation products derived from linoleic acid, and previous studies have reported higher linoleic acid concentrations in DM than in HM [48,49,50]. Similarly, heptanal, a volatile oxidation product of polyunsaturated fatty acids, also showed higher concentrations in DM, in agreement with reports indicating higher PUFA content in DM relative to HM [14,51]. These findings align with a previous study indicating that the VOCs present in meat exhibit species-specific characteristics and are closely associated with the composition of fatty acids [47]. This study employed gas chromatography–ion mobility spectrometry (GC-IMS) coupled with a multivariate statistical analysis, which presents certain methodological limitations. Future research would benefit from integrating gas chromatography–olfactometry–mass spectrometry (GC-O-MS) with advanced machine learning algorithms to comprehensively characterize food flavor profiles. The synergistic application of machine learning techniques and flavor metabolomics (flavoromics) offers significant potential to elucidate the complex relationships between food flavor compounds, chemical composition, and sensory perception. This integrated approach would enable more accurate and objective evaluation of food flavor attributes, potentially establishing new paradigms in food flavor research and sensory science.

## 5. Conclusions

Our study employed GC-IMS integrated with multivariable statistical methods to examine the VOC profiles of DM and HM. The 39 VOCs were distinguished in DM and HM, classified into five primary categories; 16 characteristic flavor compounds were screened, mainly including 1-octen-3-ol, 3-hexanone, and heptanol. Among them, 28 distinct VOCs were detected as candidate biomarkers for differentiating DM and HM, mainly including (E)-2-hexenal, (E)-2-octenal, and (E)-hept-2-enal. Altogether, our results not only provide detailed volatile profiles for both meat types, but also highlight specific flavor compounds and key discriminatory VOCs, contributing to a deeper understanding of the flavor characteristics of DM and HM. The above results provide technical support for the adulteration and identification of DM.

## Figures and Tables

**Table 1:** Volatile compounds in donkey and horse meat.

Count	Compound	CAS #	Formula	MW	RI	Rt [s]	Dt [a.u.]
1	Octanal-M	C124130	C8H16O	128.20	1014.50	603.17	1.42
2	Octanal-D	C124130	C8H16O	128.20	1014.50	603.17	1.82
3	2-pentyl furan	C3777693	C9H14O	138.20	994.70	573.84	1.25
4	Oct-1-en-3-ol-M	C3391864	C8H16O	128.20	985.70	553.45	1.16
5	Oct-1-en-3-ol-D	C3391864	C8H16O	128.20	984.30	550.47	1.60
6	Unidentified 1	unidentified	*	0.00	989.70	562.37	1.43
7	Heptanol	C53535334	C7H16O	116.20	977.40	535.60	1.40
8	Benzaldehyde	C100527	C7H6O	106.10	963.50	506.70	1.14
9	(E)-hept-2-enal-M	C18829555	C7H12O	112.20	959.30	498.20	1.25
10	(E)-hept-2-enal-D	C18829555	C7H12O	112.20	959.50	498.62	1.66
11	Dihydro-2(3h)-furanone	C96480	C4H6O2	86.10	924.60	433.93	1.08
12	Heptanal-M	C111717	C7H14O	114.20	903.40	398.66	1.34
13	Heptanal-D	C111717	C7H14O	114.20	904.10	399.80	1.69
14	2-heptanone-M	C110430	C7H14O	114.20	891.20	379.89	1.27
15	2-heptanone-D	C110430	C7H14O	114.20	892.00	381.02	1.62
16	n-Hexanol-M	C111273	C6H14O	102.20	873.20	355.42	1.33
17	n-Hexanol-D	C111273	C6H14O	102.20	871.90	353.72	1.64
18	(E)-2-hexenal-M	C6728263	C6H10O	98.10	853.50	330.40	1.18
19	(E)-2-hexenal-D	C6728263	C6H10O	98.10	851.60	328.12	1.51
20	Hexanal-M	C66251	C6H12O	100.20	796.70	267.82	1.27
21	Hexanal-D	C66251	C6H12O	100.20	792.70	263.84	1.55
22	2-Hexanone	C591786	C6H12O	100.20	782.10	253.59	1.19
23	3-Hexanone	C589388	C6H12O	100.20	783.00	254.45	1.17
24	Pentan-1-ol-M	C71410	C5H12O	88.10	765.70	237.81	1.26
25	Pentan-1-ol-D	C71410	C5H12O	88.10	765.70	237.81	1.52
26	3-hydroxybutan-2-one	C513860	C4H8O2	88.10	716.80	196.37	1.07
27	Propanoic acid-M	C79094	C3H6O2	74.10	704.20	186.96	1.11
28	Propanoic acid-D	C79094	C3H6O2	74.10	703.60	186.51	1.27
29	Unidentified 2	unidentified	*	0.00	687.10	175.27	1.12
30	Unidentified 3	unidentified	*	0.00	687.10	175.27	1.32
31	Pentanal-M	C110623	C5H10O	86.10	701.40	184.92	1.20
32	Pentanal-D	C110623	C5H10O	86.10	703.60	186.50	1.42
33	2-methyl-1-propanol	C78831	C4H10O	74.10	646.50	159.12	1.18
34	2-Butanone-M	C78933	C4H8O	72.10	591.00	139.46	1.06
35	2-Butanone-D	C78933	C4H8O	72.10	587.30	138.24	1.24
36	Nonanal	C124196	C9H18O	142.20	1105.50	749.04	1.49
37	(E)-2-octenal	C2548870	C8H14O	126.20	1068.50	685.86	1.33
38	Benzene acetaldehyde	C122781	C8H8O	120.20	1044.60	647.93	1.26
39	Unidentified 4	unidentified	*	0.00	688.70	175.97	1.36
40	Pent-1-en-3-ol	C616251	C5H10O	86.10	677.80	171.44	0.94
41	Methyl isobutyl ketone	C108101	C6H12O	100.20	732.20	208.62	1.17
42	Hexanoic acid	C142621	C6H12O2	116.20	992.10	567.77	1.31
43	Benzaldehyde	C100527	C7H6O	106.10	963.60	506.91	1.46

RI, retention indices. Rt, retention time. Dt, drift time. MW, molecular weight. #, registration number. *, unidentified.

**Table 2:** Differential volatile components in donkey and horse meat (μg/kg).

Count	Name	Class	DM	HM	log2FC	*p* Value	VIP
1	(E)-2-hexenal-D	Aldehyde	1.47 ± 0.13 a	0.48 ± 0.04 b	1.61	0.0000	1.10
2	(E)-2-hexenal-M	Aldehyde	7.71 ± 0.72 a	2.88 ± 0.29 b	1.42	0.0001	1.06
3	(E)-2-octenal	Aldehyde	2.44 ± 0.26 a	0.48 ± 0.04 b	2.33	0.0000	1.14
4	(E)-hept-2-enal-D	Aldehyde	6.96 ± 0.69 a	1.01 ± 0.08 b	2.79	0.0000	1.15
5	(E)-hept-2-enal-M	Aldehyde	26.39 ± 2.27 a	4.66 ± 0.75 b	2.50	0.0000	1.14
6	2-Butanone-D	Ketone	10.50 ± 1.11 a	4.28 ± 0.43 b	1.30	0.0001	1.07
7	2-Hexanone	Ketone	5.02 ± 0.35 a	2.48 ± 0.17 b	1.02	0.0000	1.08
8	2-heptanone-D	Ketone	12.92 ± 1.50 a	1.40 ± 0.32 b	3.21	0.0000	1.14
9	2-heptanone-M	Ketone	19.91 ± 1.66 a	5.99 ± 0.87 b	1.73	0.0000	1.11
10	2-methyl-1-propanol	Alcohol	29.76 ± 2.73 a	10.93 ± 0.79 b	1.45	0.0000	1.09
11	2-pentyl furan	Furan	12.67 ± 1.74 a	2.03 ± 0.40 b	2.64	0.0000	1.12
12	Heptanal-D	Aldehyde	18.61 ± 1.74 a	1.85 ± 0.37 b	3.33	0.0000	1.16
13	Heptanal-M	Aldehyde	20.27 ± 1.36 a	5.75 ± 0.93 b	1.82	0.0000	1.12
14	Heptanol	Alcohol	3.98 ± 0.29 a	1.18 ± 0.07 b	1.76	0.0000	1.13
15	Hexanal-D	Aldehyde	229.16 ± 15.15 a	79.20 ± 11.79 b	1.53	0.0000	1.11
16	Nonanal	Aldehyde	5.19 ± 0.43 a	1.86 ± 0.13 b	1.48	0.0000	1.11
17	Octanal-D	Aldehyde	4.30 ± 0.38 a	1.08 ± 0.09 b	2.00	0.0000	1.12
18	Octanal-M	Aldehyde	11.72 ± 0.83 a	3.54 ± 0.31 b	1.73	0.0000	1.14
19	Pentanal-D	Aldehyde	49.83 ± 3.92 a	3.27 ± 0.88 b	3.93	0.0000	1.16
20	Pentanal-M	Aldehyde	39.27 ± 2.57 a	9.12 ± 2.23 b	2.11	0.0008	1.10
21	Benzene acetaldehyde	Aldehyde	2.76 ± 0.28 a	0.46 ± 0.06 b	2.58	0.0000	1.14
22	Hexanoic acid	Acid	3.30 ± 0.37 a	1.22 ± 0.14 b	1.43	0.0002	1.04
23	n-Hexanol-D	Alcohol	17.59 ± 0.80 a	2.11 ± 0.42 b	3.06	0.0000	1.16
24	n-Hexanol-M	Alcohol	35.83 ± 2.07 a	10.98 ± 1.54 b	1.71	0.0000	1.13
25	Oct-1-en-3-ol-D	Alcohol	19.55 ± 1.78 a	3.94 ± 0.73 b	2.31	0.0000	1.12
26	Oct-1-en-3-ol-M	Alcohol	74.50 ± 5.48 a	25.44 ± 3.56 b	1.55	0.0000	1.11
27	Pentan-1-ol-D	Alcohol	78.63 ± 7.00 a	13.68 ± 2.51 b	2.52	0.0000	1.14
28	Pentan-1-ol-M	Alcohol	74.70 ± 4.74 a	37.70 ± 3.31 b	0.99	0.0001	1.07
29	Unidentified 1	Unidentified	44.91 ± 3.88 a	14.83 ± 2.44 b	1.60	0.0001	1.09
30	Unidentified 4	Unidentified	28.76 ± 2.30 a	8.15 ± 0.76 b	1.82	0.0000	1.14

a,b: Values of a and b with different letters in the same row indicate significant differences. VIP, variable importance in projection. DM, donkey meat. HM, horse meat. FC in log2FC, i.e., fold change, indicates the ratio of expression between two samples (groups).

## Data Availability

The original contributions presented in this study are included in the article. Further inquiries can be directed to the corresponding authors.
